# Induction of Viable but Nonculturable State in *Rhodococcus* and Transcriptome Analysis Using RNA-seq

**DOI:** 10.1371/journal.pone.0147593

**Published:** 2016-01-25

**Authors:** Xiaomei Su, Li Guo, Linxian Ding, Kun Qu, Chaofeng Shen

**Affiliations:** 1 Department of Environmental Engineering, College of Environmental and Resource Sciences, Zhejiang University, Hangzhou 310058, China; 2 Key Laboratory for Water Pollution Control and Environmental Safety in Zhejiang Province, Hangzhou 310058, China; 3 College of Geography and Environmental Science, Zhejiang Normal University, Jinhua 321004, China; 4 School of Medicine, Stanford Universtiy, Stanford, California 94305, USA; Dong-A University, REPUBLIC OF KOREA

## Abstract

Viable but nonculturable (VBNC) bacteria, which maintain the viability with loss of culturability, universally exist in contaminated and non-contaminated environments. In this study, two strains, *Rhodococcus* sp. TG13 and TN3, which were isolated from PCB-contaminated sediment and non-contaminated sediment respectively, were investigated under low temperature and oligotrophic conditions. The results indicated that the two strains TG13 and TN3 could enter into the VBNC state with different incubation times, and could recover culturability by reversal of unfavourable factors and addition of resuscitation-promoting factor (Rpf), respectively. Furthermore, the gene expression variations in the VBNC response were clarified by Illumina high throughput RNA-sequencing. Genome-wide transcriptional analysis demonstrated that up-regulated genes in the VBNC cells of the strain TG13 related to protein modification, ATP accumulation and RNA polymerase, while all differentially expressed genes (DEGs) in the VBNC cells of the strain TN3 were down-regulated. However, the down-regulated genes in both the two strains mainly encoded NADH dehydrogenase subunit, catalase, oxidoreductase, which further verified that cold-induced loss of ability to defend oxidative stress may play an important role in induction of the VBNC state. This study further verified that the molecular mechanisms underlying the VBNC state varied with various bacterial species. Study on the VBNC state of non-pathogenic bacteria will provide new insights into the limitation of environmental micro-bioremediation and the cultivation of unculturable species.

## Introduction

The genus *Rhodococcus* plays a significant role in the fields of environmental bioremediation and industrial biotechnology [1]. The pollutant-degrading abilities of many species have been widely used for the biodegradation of xenobiotic compounds which are recalcitrant with remarkable stability and toxicity, including various herbicides, nitroaromatics, chloroaromatics and polychlorinated biphenyls (PCBs) [2]. Various bacterial strains of the genus *Rhodococcus* represent an abundant part of indigenous bacterial communities in contaminated localities [3]. The versatile degradative potential of *Rhodococcus* species and their ability to persist in adverse conditions make them suitable industrial microorganisms for bioremediation of contaminated sites. In addition, there has been increased interest in potential health risks caused by pathogenic rhodococci in humans, animals and plants [4]. Therefore, isolating and culturing difficult-to-culture or VBNC bacteria, searching for new *Rhodococcus* species, and understanding their related function and genotype have become increasingly more important.

However, to date, just over 40 species with validly published names in the genus *Rhodococcus* have been described [5], because most of bacteria in natural environments cannot be cultivated. It is very common for bacteria to survive by entering into a “viable but non-culturable” (VBNC) state [6, 7] when they encounter harsh environmental conditions including nutrient starvation, extreme temperature, elevated or lowered osmotic concentrations, copper stress and even exposure to persistent organic pollutants [8]. Specifically, *Rhodococcus* species, such as *R*. *rhodochrous* and *R*. *fascians* are known to enter into the VBNC state under adverse conditions [9]. Bacteria in the VBNC state exhibit various modifications, which include size reduction, metabolic activity decrease, and changes in cell membrane composition and cell wall structure [10]. Up to now, approximately 70 bacterial species described to enter the VBNC state, and the number of species constantly increases. Most of studies characterized the formation and resuscitation of the VBNC bacteria, including *Vibrio*, *Salmonella*, *Legionella* and *Escherichia* [10–12], due to their infectious and pathogenic potentials [7, 8], there is hardly any information concerning the environmental functions and nature resource underlying the VBNC bacteria [6].

Meanwhile, most of studies elucidated the changes of VBNC state with regard to the physiological and biochemical aspects [11, 12]. A few studies have examined the genetic modulation involved in induction of entry and exit from the VBNC state. Coutard et al. [13] demonstrated that the recovery of culturability of VBNC *Vibrio parahaemolyticus* has no direct correlation with the induction of virulence gene expression by a real-time reverse transcription-PCR method. Asakura et al. [14] evaluated the changes in gene expression of VBNC *Vibrio cholerae* cells by comparative microarray analysis. Recent research has shown that the high throughput RNA-sequencing (RNA-Seq) technology, which could capture unbiased view of the RNA transcript profile of a species under a given condition at the whole genome level, is a powerful and cost-effective tool for transcriptome analysis [15]. However, little work has been performed to investigate the genetic mechanisms underlying the VBNC state by using high throughput RNA-Seq.

It is worth to note that the genes expressed during entering the VBNC state which differs from bacterium to bacterium are complex and far from being fully understood. Although gene expression and the proteins modulation of VBNC cells have been studied, the molecular mechanism underlying the VBNC state is still poorly understood [16]. It has been recognized for many years that the global stress regulator, *rpo*S (σ^S^) is required for the survival of numerous bacteria in multiple-stress environment [17, 18]. Moreover, the concentration of guanosine 3’,5’-bispyrophosphate (ppGpp) depends upon the activity of two proteins RelA and SpoT. The up-regulation of the *rel*A and *spo*T genes during entering the VBNC state would lead to accumulation of ppGpp, followed by accumulation of σ^S^, ultimately leading to enhanced stress resistance [10]. In addition, cold-induced loss of ability to defend oxidative stress may play a role in induction of the VBNC state [18–20]. Wang et al. [21] demonstrated that the antioxidative activities of alkyl hydroperoxide reductase subunit C (AhpC) proteins which are members of a family of peroxidases played a significant role in the induction and maintenance of the VBNC state in *V*. *parahaemolyticus*. It is important to stress that some VBNC bacteria are able to recover culturable state when surrounding conditions are more favorable [7], but some need to resuscitate by resuscitation-promoting factor (Rpf) which is secreted by *Micrococcus luteus* [22]. Rpf can promote the resuscitation and growth of high G+C Gram-positive organisms which contain well-known pollutant-degraders, including *Rhodococcus*, *Mycobacterium*, *Arthrobacter* [23, 24]. Moreover, the addition of supernatant from growing *M*. *luteus* cultures to VBNC cells has been shown to restore their culturability [9].

In our previous study, many uncultured or VBNC bacteria were obtained from PCB-contaminated soils and sediments by adding EOM from *M*. *luteus* [23, 25]. Notably, a novel species of *Rhodococcus biphenylivorans* were obtained and could be entered into the VBNC state under low temperature and oligotrophic conditions [23, 26]. Therefore, a broader understanding of the VBNC bacteria of genus *Rhodococcus* in contaminated and non-contaminated environments could help us to reveal why the activities of pollutant-degrading bacteria decreased in the pilot-scale environmental bioremediation, and provide new insights into control strategies for prevention and resuscitation of bacteria from the VBNC state, for the missing species into culture. In this work, the induction of the VBNC state in two *Rhodococcus* sp. strains TG13 and TN3 which were isolated from PCB-contaminated sediment and non-contaminated sediment respectively, were performed under oligotrophic and low temperature conditions. A comparison of the ability of cells to exit the VBNC state was also investigated. Most notably, the primary purpose of the present study was to access the gene expression variation and regulation underline the VBNC state. In comparison to the normal cells, the differentially expressed genes (DEGs) in the VBNC cells were identified by high throughput RNA-Seq. The functions and related metabolic pathways of the up- or down-regulated genes were investigated by Gene Ontology (GO) and Kyoto Encyclopedia of Genes and Genomes (KEGG) enrichment analysis. A comprehensive comparison of the genetic mechanisms involved in the VBNC state of the two *Rhodococcus* strains was performed. To our best knowledge, it is the first attempt to compare the VBNC response of two *Rhodococcus* sp. strains which one of them possesses biphenyl/PCB-degradation capability.

## Materials and Methods

### Ethics Statement

No specific permissions were required for the described field studies.

### Bacterial strains and growth conditions

Two strains, *Rhodococcus* sp. TG13 and TN3, which were obtained by using the resuscitation function of EOM from *M*. *luteus*, were used in this study. The two strains were isolated from PCB-contaminated river sediment and non-contaminated deep sea sediment respectively, in Taizhou city, Zhejiang province, China. The two strains have been deposited in Marine Culture Collection of China (MCCC) with deposit numbers 1K01234 and 1K01235. Meanwhile, the 16S rDNA sequences of the two strains have been submitted to NCBI GenBank under accession numbers KM235731 and KP410397. The two strains TG13 and TN3 shared highest similarities (99.3–100% 16S rRNA gene similarity) with *Rhodococcus ruber* (100% similarity) and *Rhodococcus erythropolis* (99.3% similarity), respectively. The pure strains were stored in Luria-Bertani (LB) broth with 20% glycerol at -80°C. The strains were incubated in LB medium at 30°C with shaking at 180 rpm and grew to the exponential phase (OD_600_ = 0.98).

### Conditions inducing the VBNC state

Bacterial cell cultures in the exponential-phase were therefore harvested by centrifuged (8000 g, 15 min) and washed twice with 0.85% (w/v) sterile NaCl solution (pH = 7.3). Then, the washed cells were resuspended in an artificial oligotrophic medium (AOM) which contained (per liter): 1 g (NH_4_)_2_SO_4_, 1 g KH_2_PO_4_, 3 g K_2_HPO_4_ ·3H_2_O, 0.2 g MgSO_4_, 0.02 g FeSO_4_·7H_2_O and 5 g NaCl, at a final cell density of 1 × 10^7^ CFU/mL (the control groups, c_TG13 and c_TN3). The resuspended cells of strains TG13 and TN3 were maintained at 4°C for 91 and 163 days, respectively to induce the VBNC state (< 0.1 CFU/mL) (the treatment groups, t_TG13 and t_TN3). All the experiments were performed in triplicate. Each sample obtained at distinct times was analyzed by using acridine orange direct counts (AODC) and flow cytometry, and plated on LB agar for culturable counts, presenting total, viable and culturable numbers of bacterial cells, respectively.

### Analysis of viability and culturability

Cell viability was assessed by using the LIVE/DEAD *Bac*Light bacterial viability kit (Molecular Probes, Inc., Eugene, OR, USA) according to the manufacturer’s instructions. Samples were added to the BD TruCOUNT^TM^ Tubes (BD Biosciences, San Diego, CA, USA), and analyzed by an Epics XL flow cytometry (Coulter Corporation, Miami, FL, USA). The fluorescence of stains SYTO 9 and propidium iodide (PI) were detected through 525 nm and 620 nm bands pass filters, respectively. In order to determine the culturability of the cells in samples, each sample was serially (1:10) diluted with 0.85% NaCl solution and then pour-plated the cells on LB agar at 30°C for 48 h. Colony-forming units were enumerated and given as counts per millilitre (CFU/mL) of the initial suspension. All these experiments were performed in triplicate. The average value and standard deviation were calculated using IBM SPSS Statistics.

### Resuscitation from VBNC state

The VBNC cells (< 0.1 CFU/mL) in this study were centrifuged at 8,000 g for 10 min, and then the pellets were resuspended in the same volume of sterile 0.85% NaCl solution. Before resuscitation, the VBNC cells were treated for 2 h with 200 mg/mL of benzylpenicillin [17]. Then, resuscitation experiments for each strain were divided into two groups including with Rpf addition (0.05%, v/v) and without Rpf addition. The cultures were incubated at 30°C in a static state for 3–4 days. The culturability was determined by plating the cells on the LB agar plates. Furthermore, most probable number (MPN) estimates were used when the resuscitation experiments were performed in liquid media, and the MPNs were calculated at 12 h intervals within 84–96 h. All experiments described here were performed in triplicates. The average value and standard deviation were calculated using IBM SPSS Statistics.

### Illumina high-throughput transcriptome sequencing

#### RNA extraction

The exponential and VBNC cells of the two strains were used for RNA-Seq analysis. All experiments were performed in duplicates. For each biological sample, total RNA samples were extracted using the MICROBExpress Bacterial mRNA Enrichment kit (Life Technologies, Grand Island, NY, USA) and then was examined with an Agilent 2100 Bioanalyzer. At least 20 μg of RNA at a concentration of ≥ 400 ng/μL, OD260/280 = 1.8–2.2 and RNA28S:18S ≥ 1.0 were used for cDNA library preparation [27].

#### cDNA library preparation for transcriptome sequencing

Illumina sequencing was performed at the Beijing Genomic Institute (BGI-Shenzhen, China). Briefly, after removing residual DNA and ribosomal RNA, the mRNA-enriched RNA was chemically fragmented to 150~200 bp. Based on these cleaved RNA fragments, cDNA was synthesized using random hexamer primer and reverse transcriptase. After the end reparation and ligation of adaptors, the products were amplified by PCR and purified using the QIAquick PCR Purification Kit (Qiagen, Valencia, CA, USA) to create the final cDNA library. Libraries were sequenced on an Illumina HiSeq^TM^ 2000 platform [28].

#### Mapping reads to the reference genome

Image data output from the sequencing machine is transformed into raw reads and stored in FASTQ format. All the raw transcriptome data have been deposited in the NCBI Short Read Archive (SRA), and given the accession numbers SRA236830 and SRA236842 for the strain TG13 and TN3, respectively. These dirty raw reads (reads with adaptors, reads with unknown base more than 5%, or low quality reads) were removed to obtain clean reads. Subsequently, the clean reads of the strains TG13 and TN3 were mapped to reference genome of *R*. *ruber* (http://www.ncbi.nlm.nih.gov/genome/11562?genome_assembly_id=217613) and *R*. *erythropolis* PR4 (http://www.ncbi.nlm.nih.gov/genome/1638?genome_assembly_id=171453) using BWA-MEM alignment [29]. Mismatches no more than 5 bases were allowed in the alignment.

#### Analysis of gene expression variations

Gene expression counts in the control groups (c_TG13 and c_TN3) and the treatment groups (t_TG13 and t_TN3) were obtained using featureCounts software (version 1.4.6) which was set to exclude reads or fragments overlapping multiple genes. The values of normalized mean count obtained from the average value of the biological replicate experiments. The tool EBseq (version 1.7.1) was used to identify the DEGs between exponential and VBNC cells. To determine the statistical significant difference in gene expression, an absolute value of log2 ratio ≥ 1 and a false discovery rate (FDR) ≤ 0.05 were used as the threshold [30].

For the DEGs, GO enrichment analyses were performed using a GO seq-based Wallenius non-central hyper-geometric distribution [31]. Furthermore, the KEGG pathway enrichment analysis of DEGs was also performed to identify significantly enriched metabolic pathways using the KEGG Orthology (KO) system [32]. After multiple testing correction, the corrected P-value < 0.05 was selected as a threshold to determine the significant enrichment of DEGs.

### Quantitative real-time RT-PCR (qRT-PCR) validation

DEGs were randomly selected for qRT-PCR to prove the quality of the sequencing data. The mRNAs were extracted as described for the cDNA library preparation, and then were transcribed to cDNA using SuperScript III reverse transcriptase. Subsequently, the qRT-PCR was performed using a power SYBR Green PCR kit (Applied Biosystems, Foster, CA, USA) in a MicroAmp™ 96-well plate with the ABI 7500 real-time PCR system. Gene-specific primers used for qRT-PCR are shown in S1 Table. The V3 variable region of 16S rRNA was served as an internal standard, and the 16S rRNA gene universal primers 8F (5’-AGAGTTTGATCCTGGCTCAG-3’) and 1541R (5’-AAGGAGGTGATCCAGCCGCA-3’) was used for PCR amplification. The normalized fold changes of the relative expression ratio were quantified by the 2^-ΔΔCT^ method [33]. All experiments were performed in triplicates and their average values were calculated using IBM SPSS Statistics.

## Results

### Evidence for entering a VBNC state

**Fig 1:**
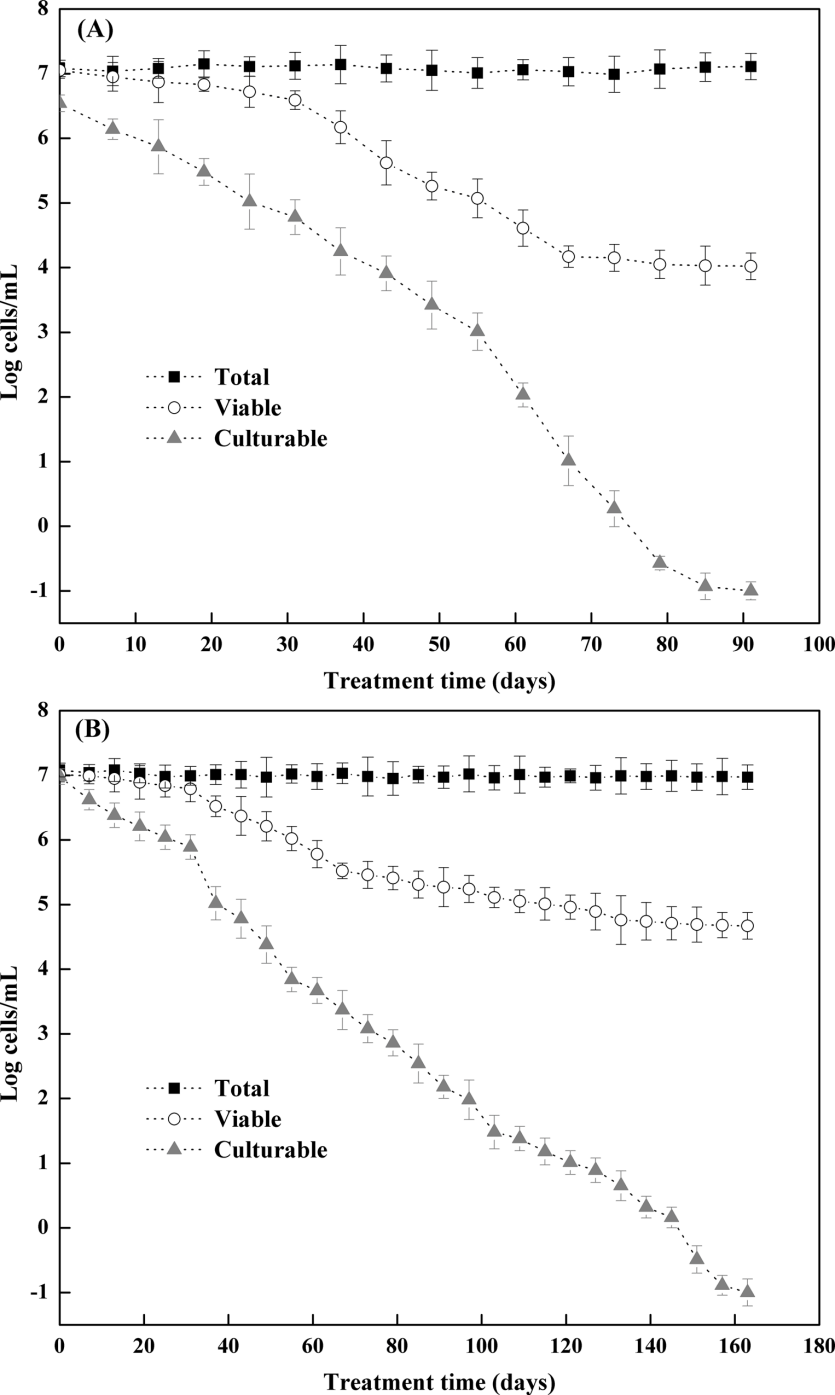
Evidence for *Rhodococcus* sp. strains TG13 (A) and TN3 (B) entry into the VBNC state in an artificial oligotrophic medium at 4°C, as determined by AODC (■), FCM (○) and plate counting (▲) methods. Error bars show the standard deviations of triplicate experiments.

Cell viability of the strains TG13 and TN3 was assessed once a week. After 91 and 163 days of incubation at 4°C in AOM, the number of culturable cells of the strains TG13 (Fig 1A) and TN3 (Fig 1B) counted by plating decreased to undetectable levels (< 0.1 CFU/mL), while viable cell counts of the strains TG13 and TN3 showed a low decrease, revealing approximated 10^4^ and 10^5^ cells/mL, respectively. And total cell counts remained constant at the initial level (10^7^ cells/mL). These results demonstrated that the strains TG13 and TN3 could enter the VBNC state under the present conditions. Moreover, the entry into the VBNC cells was also verified by resuscitation experiments.

### Resuscitation from the VBNC state

**Fig 2:**
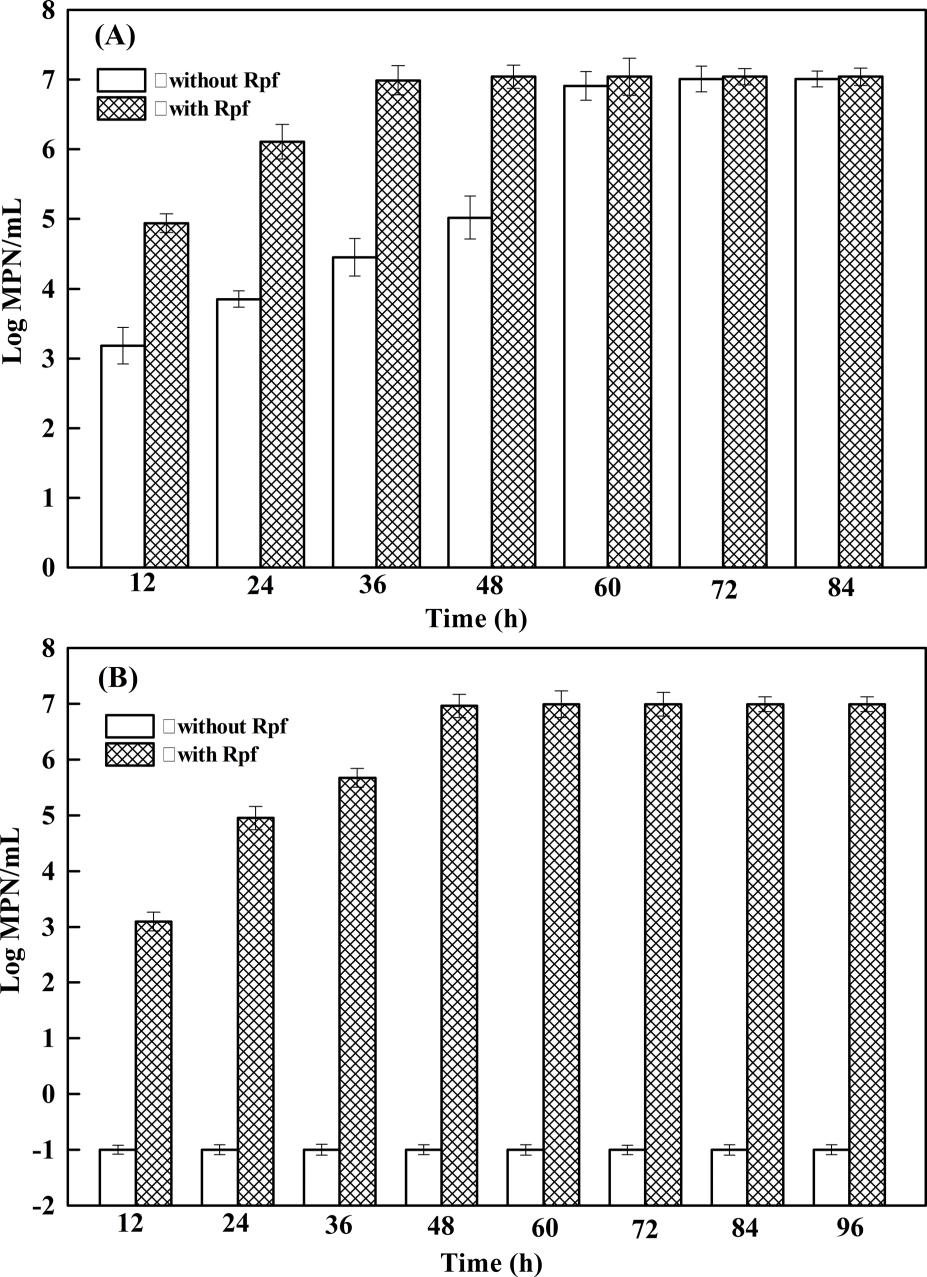
Measuring the resuscitated cells *Rhodococcus* sp. strains TG13 (A) and TN3 (B) by MPN method. Error bars show the standard deviations of triplicate experiments.

Before resuscitation, the VBNC cells were treated with benzylpenicillin to overcome the argument that the observed resuscitation due to the regrowth of still culturable cells existed in the VBNC microcosm. As shown in Fig 2A, for the strain TG13, the MPNs of the resuscitable cells without Rpf addition rose to 7.08 × 10^3^ MPNs/mL at 24 h and reached a peak value (1.02 × 10^7^ MPNs/mL) at 72 h. After 72 h, the cells entered stationary phase, which indicated that the increased value after 24 h was caused by the multiplication of the resuscitated cells. However, with Rpf addition, the VBNC cells could be resuscitated and multiplied to 8.71 × 10^4^ MPNs/mL within 12 h. This comparison indicated that Rpf only stimulated resuscitation of the strain TG13, and the VBNC cells of the strain TG13 could resuscitate without Rpf addition. For strain TN3 (Fig 2B), it could not resuscitate without Rpf addition. The MPNs of the resuscitation maintained undetectable levels after growing in LB medium for 96 h without Rpf addition, while the MPNs of the resuscitable cells with Rpf addition increased to 8.91 × 10^4^ MPNs/mL at 24 h, and reached a peak value (9.77 × 10^6^ MPNs/mL) at 60 h by cells multiplication. Moreover, the above described results were also verified by resuscitation performed with solid media. Colonies of the strain TG13 appeared when plating the VBNC cells on the LB agar plates at 30°C for 3 days, while the VBNC cells of strain TN3 could not grow under the same condition. These results indicated that for resuscitation from VBNC state, the strain TN3 needed extracellular bacterial proteins, but the strain TG13 could be achieved by a simple reversal of the unfavourable factors.

### Comprehensive screening of VBNC-responsive genes

Two cDNA libraries for the strains TG13 and TN3 were constructed, respectively. As shown in Table 1, a total of 19,908,800, 20,163,796, 23,007,580 and 21,442,376 raw reads from cDNA libraries of c_TG13, t_TG13, c_TN3 and t_TN3 were generated, respectively. For the strain TG13, 75% and 51% clean reads obtained from c_TG13 and t_TG13 samples, respectively, were mapped to *R*. *ruber* genome. For the strain TN3, 76% and 70% of obtained reads in the c_TN3 and t_TN3, respectively, were covered in *R*. *erythropolis* PR4 genome. It is worth noting that total reads which were perfectly mapped to the reference genomes without mismatch were all greater than 50%. All these results indicated that the obtained sequences met the requirement, and could be used for further analysis.

**Table 1:** An overview of the RNA-Seq statistics.

	Strain TG13		Strain TN3	
	c_TG13	t_TG13	c_TN3	t_TN3
	Number (Percentage)	Number (Percentage)	Number (Percentage)	Number (Percentage)
**Total raw reads**	19908800	20163796	23007580	21442376
**Total raw base pairs**	1791792000	1814741640	2070682200	1929813840
**Total clean reads**	1697090410	1717148293	1959514539	1821156054
**Total clean base pairs**	1697090410	1717148293	1959514539	1821156054
**Reads mapping to genome**	14539154 (75%)	10023120 (51%)	17199416 (76%)	14495968 (70%)
**Assigned to gene**	8141934 (41.79%)	3692364 (18.82%)	13810066 (61.34%)	6601058 (31.71%)

**Fig 3:**
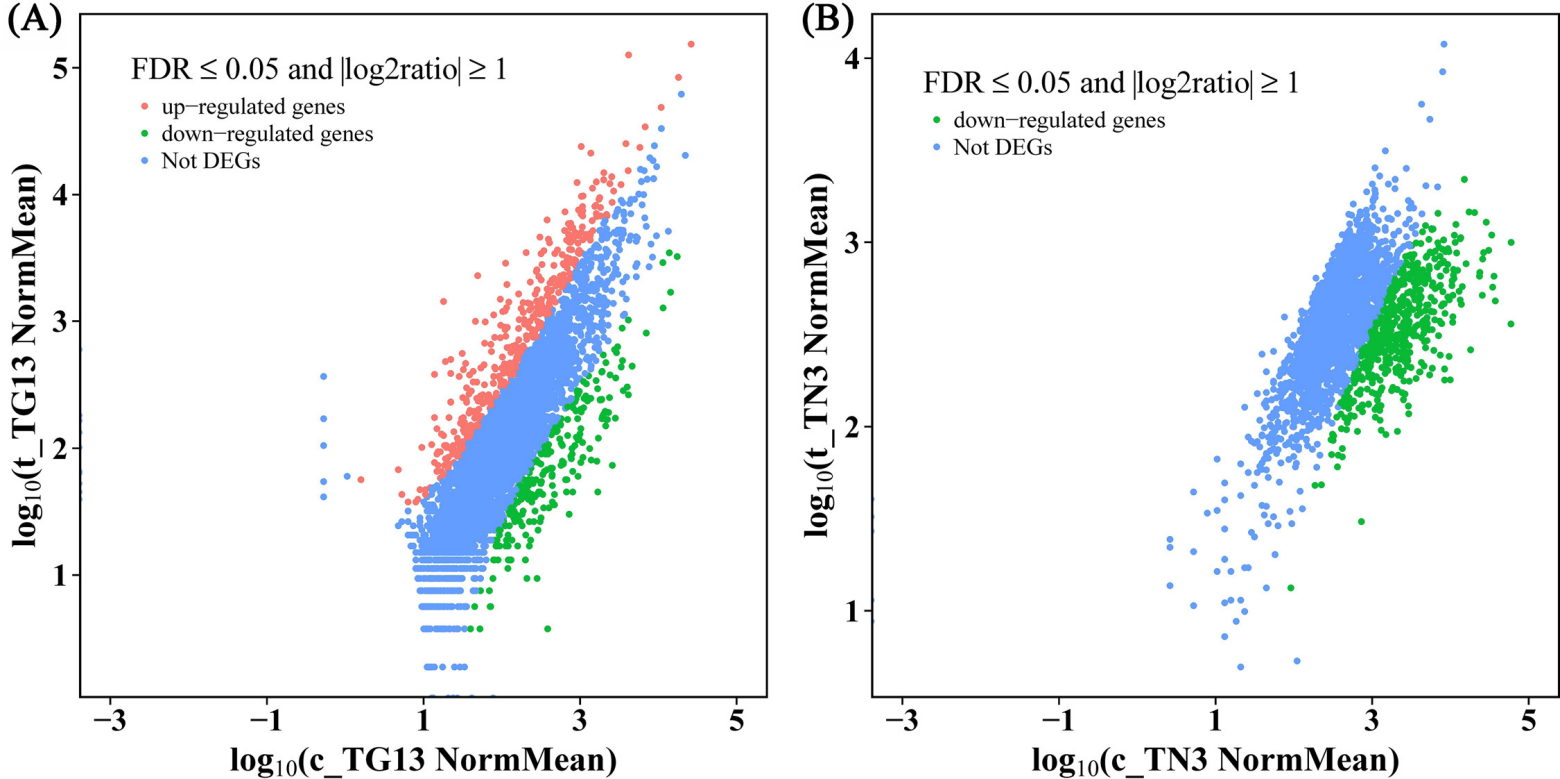
Differential expression levels of the exponential-phase cells and VBNC cells in *Rhodococcus* sp. strains TG13 (A) and TN3 (B). The absolute value of log2 ratio ≥ 1 and the false discovery rate (FDR) ≤ 0.05 were used as the threshold.

Gene expression of t_TG13 and t_TN3 in VBNC state were compared to that of c_TG13 and c_TN3 in exponential growth stage, respectively (S2 Table). A total of 4720 and 5502 predicted genes were expressed in TG13 and TN3, respectively (S2 Table). And most gene IDs (4697 of 4720 and 5463 of 5502) were annotated by the NR database. To determine which genes were differentially expressed during VBNC state formation, |log2 ratio| ≥ 1 and FDR ≤ 0.05 were set as the threshold to assess the statistical significance of the differential gene expression. As shown in Fig 3 and S3 Table, for the strain TG13, a total of 634 DEGs were detected, including 391 up-regulated genes and 243 down-regulated genes. While for the strain TN3, 992 DEGs were detected and all of them were down-regulated. As listed in S4 Table, a total of 17 and 95 genes were differentially expressed more than 20-fold in the strains TG13 and TN3, respectively. 10 of the 17 DEGs were up-regulated and 7 were down-regulated in the strain TG13. In contrast, all the 95 DEGs were down-regulated in the strain TN3, and of which 36 DEGs were differentially expressed more than 40-fold.

### GO functional analysis of DEGs

**Fig 4:**
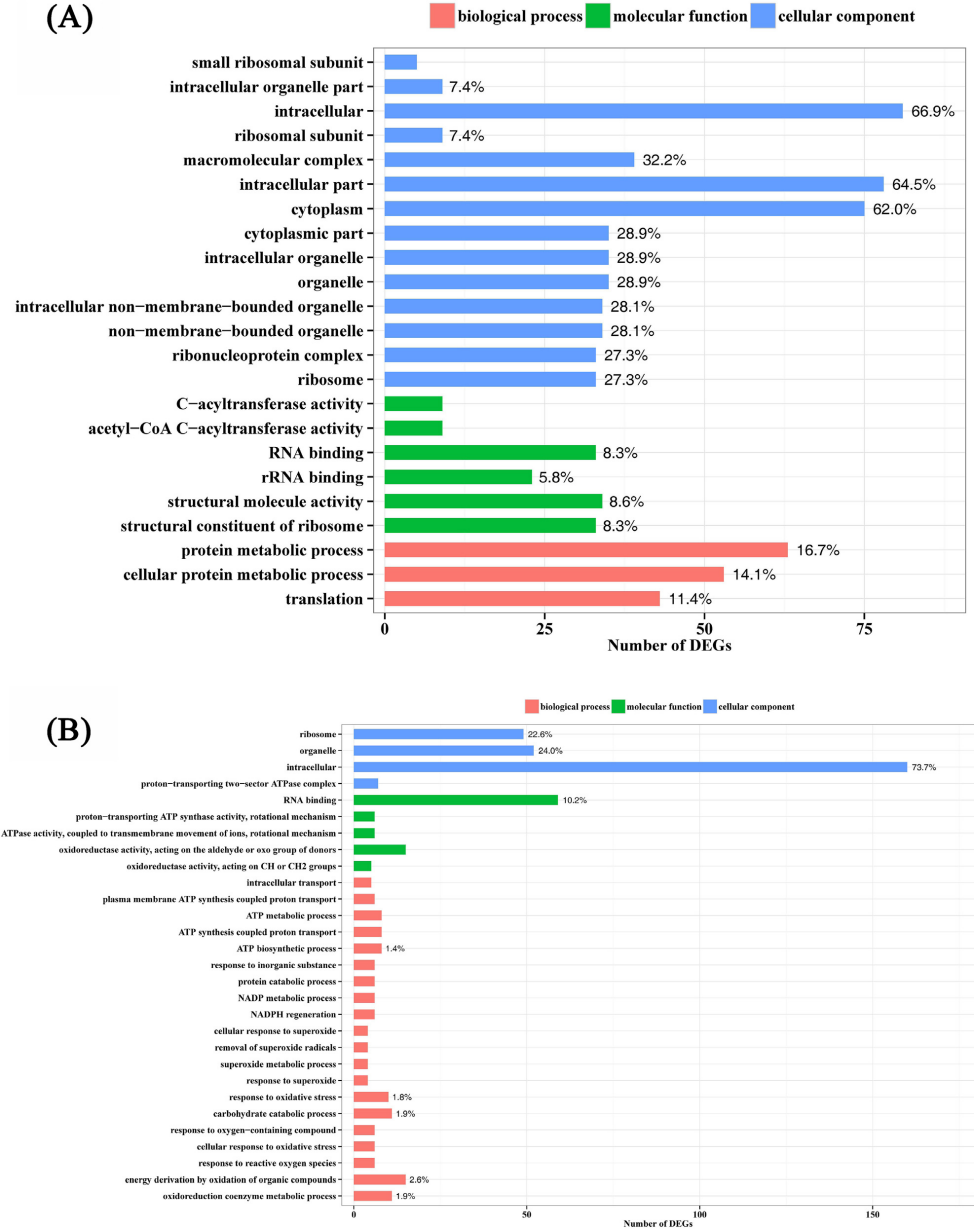
Significant enriched GO terms of differentially expressed genes in *Rhodococcus* sp. strains TG13 (A) and TN3 (B). The y-axis denotes the number of genes in a category. The number above the bar denotes the percentage of a specific term of genes in the main category.

To understand the function of the DEGs underlying the VBNC state, GO enrichment analysis was performed with the total of 634 and 992 DEGs in the strains TG13 and TN3, respectively. Based on sequence homology, DEGs of the strains TG13 and TN3 were assigned to one or more GO codes and categorized into 1031 and 1478 secondary level GO terms (S5 Table), respectively. In each of the three main categories (biological process, molecular function and cellular component) of the GO classification, there were 551, 434, and 46 functional groups in the strain TG13, respectively, and were 824, 590, and 64 functional groups in the strain TN3, respectively. For strain TG13, 23 GO terms were significantly enriched, of which 3, 6 and 14 GO terms in each of the three main categories (Fig 4A and S5 Table in bold). The terms of “protein metabolic process”, “structural molecule activity”, “RNA binding”, “intracellular”, “intracellular part”, “cytoplasm” and “macromolecular complex” made up the majority. To further understand the GO function of the up- or down-regulated genes in t_TG13 and c_TG13 libraries, GO enrichment analysis was performed with the 391 up-regulated and 243 down-regulated genes, respectively. As shown in S6 Table and S1 Fig, for the 805 enrichment GO terms of up-regulated genes, there were 6, 15, and 15 significantly enriched GO terms in each of the three main categories, respectively. In the 6 terms of biological process category, “protein metabolic process” was the most highly represented. The terms of “coenzyme binding”, “structural molecule activity” and “RNA binding” were dominant in molecular function category. In the category of cellular component, up-regulated genes were significantly enriched with the terms of “cell”, “cell part”, “intracellular”, “intracellular part” and “cytoplasm”. It is worth noting that the terms of “RNA polymerase activity”, and “sigma factor activity” were main functions of up-regulated gene when compared up-regulated genes with the genome genes in each term. Meanwhile, there were only 4 terms of “NADH dehydrogenase (quinone) activity”, “oxidoreductase activity”, “NADH dehydrogenase activity” and “quinone binding” significant enriched for the 513 enrichment GO terms of down-regulated genes (S6 Table in bold).

However, for strain TN3, in each of the three main categories of the GO classification, there were 138, 34 and 23 functional groups significantly enriched, respectively (S5 Table in bold). As shown in S5 Table and Fig 4B, although the function of DEGs covered a comprehensive range of GO categories, “energy derivation by oxidation of organic compounds”, “oxidoreduction coenzyme metabolic process”, “carbohydrate catabolic process”, “response to oxidative stress” and “ATP biosynthetic process” were dominant in the main category of biological process. The terms of “RNA binding” and “oxidoreductase activity” were dominant in the main category of molecular function. The terms of “intracellular”, “organelle” and “ribosome” were dominant in the main category of cellular component.

### Pathway analysis of DEGs

To identify the biological pathways that are active in the formation of the VBNC state, all of the genes were mapped to the reference canonical pathways in KEGG and compared with the whole genomic background. As shown in S7 Table, for strain TG13, of the total 1261 genes with the KEGG pathway annotation, 263 DEGs were assigned to 118 pathways. The three KEGG pathways of “citrate cycle”, “butanoate metabolism” and “synthesis and degradation of ketone bodies” were significantly enriched (bold in S7 Table). Furthermore, the three pathways mentioned above were also significant enrichment in 106 enriched KEGG pathways of up-regulated genes (bold in S8 Table). Notably, although the pathways of “RNA polymerase” and “FoxO signaling pathway” were not significantly enriched, they were main functions of up-regulated gene when compared up-regulated genes with the genome genes in each pathway. In contrast, in 67 enriched KEGG pathways of down-regulated genes, there was no significant enrichment observed. The pathway of “nitrotoluene degradation” was the greatest representation (S8 Table). However, for strain TN3, among all the 1375 genes with the KEGG pathway annotation, 340 down-regulated genes were involved in 137 pathways in which “oxidative phosphorylation” and “carbon metabolism” were significantly enriched (bold in S7 Table).

**Fig 5:**
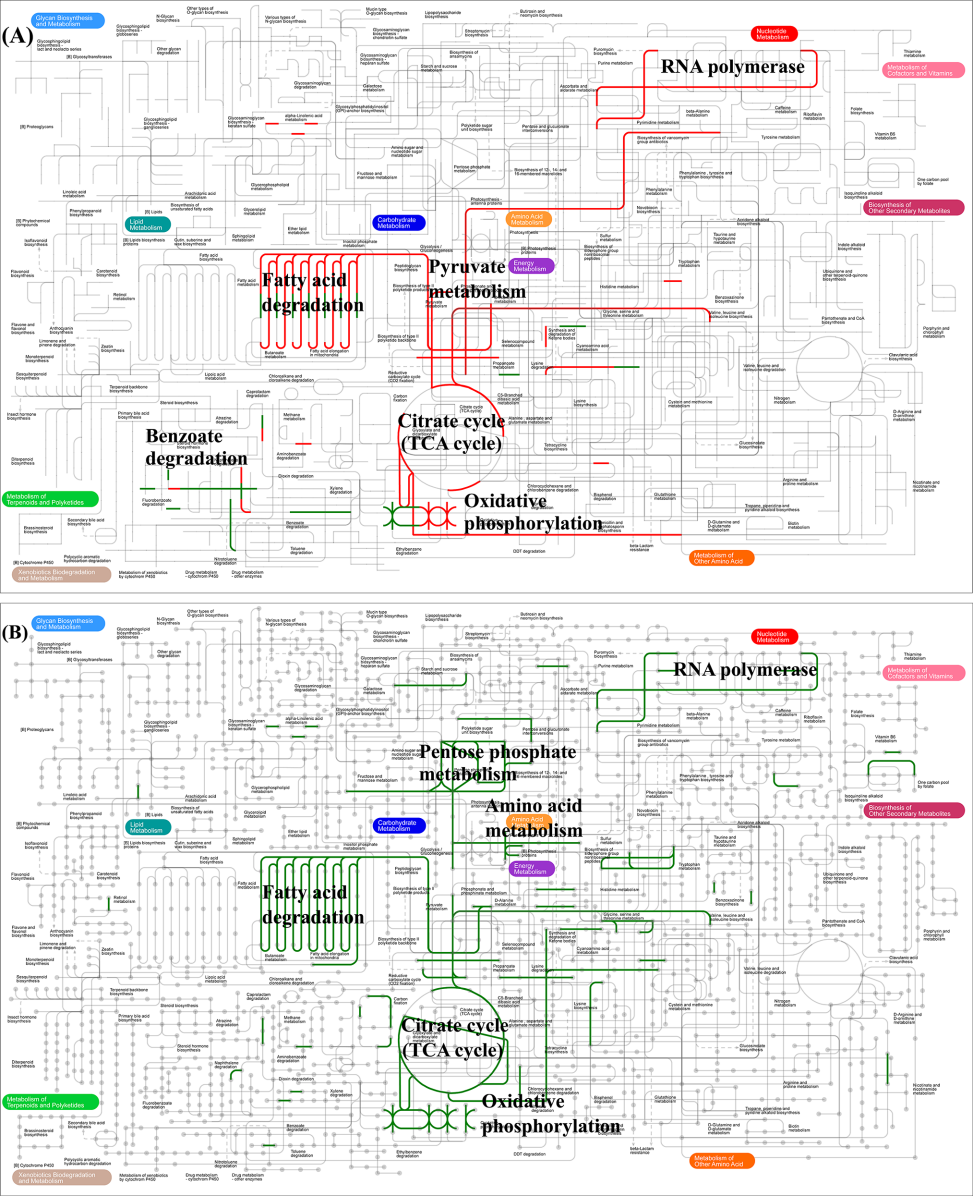
Metabolic pathway maps of the selected differentially expressed genes in *Rhodococcus* sp. strains TG13 (A) and TN3 (B). The red and the green lines indicate genes with up-regulated and down-regulated, respectively.

In addition, to provide a global view of metabolism of DEGs with expression fold change of at least 20-fold up or down-regulation (S4 Table), some DEGs were submitted for analysis via the on-line interactive pathways explorer (iPath2). For strain TG13, 76 DEGs with 56 KO IDs were submitted. As shown in Fig 5A, the mapping pathways were related to “citrate cycle”, “oxidative phosphorylation”, “RNA polymerase”, “fatty acid degradation”, “pyruvate metabolism” and “benzoate degradation”. While for strain TN3, total 123 DEGs with 111 KO IDs were submitted for analysis. As shown in Fig 5B, the mapping pathways were related to “citrate cycle”, “oxidative phosphorylation”, “RNA polymerase”, “fatty acid degradation”, “amino acid metabolism” and “pentose phosphate metabolism”. These results were in accord with the results of enrichment analysis.

### Quantitative real-time PCR validation

**Fig 6:**
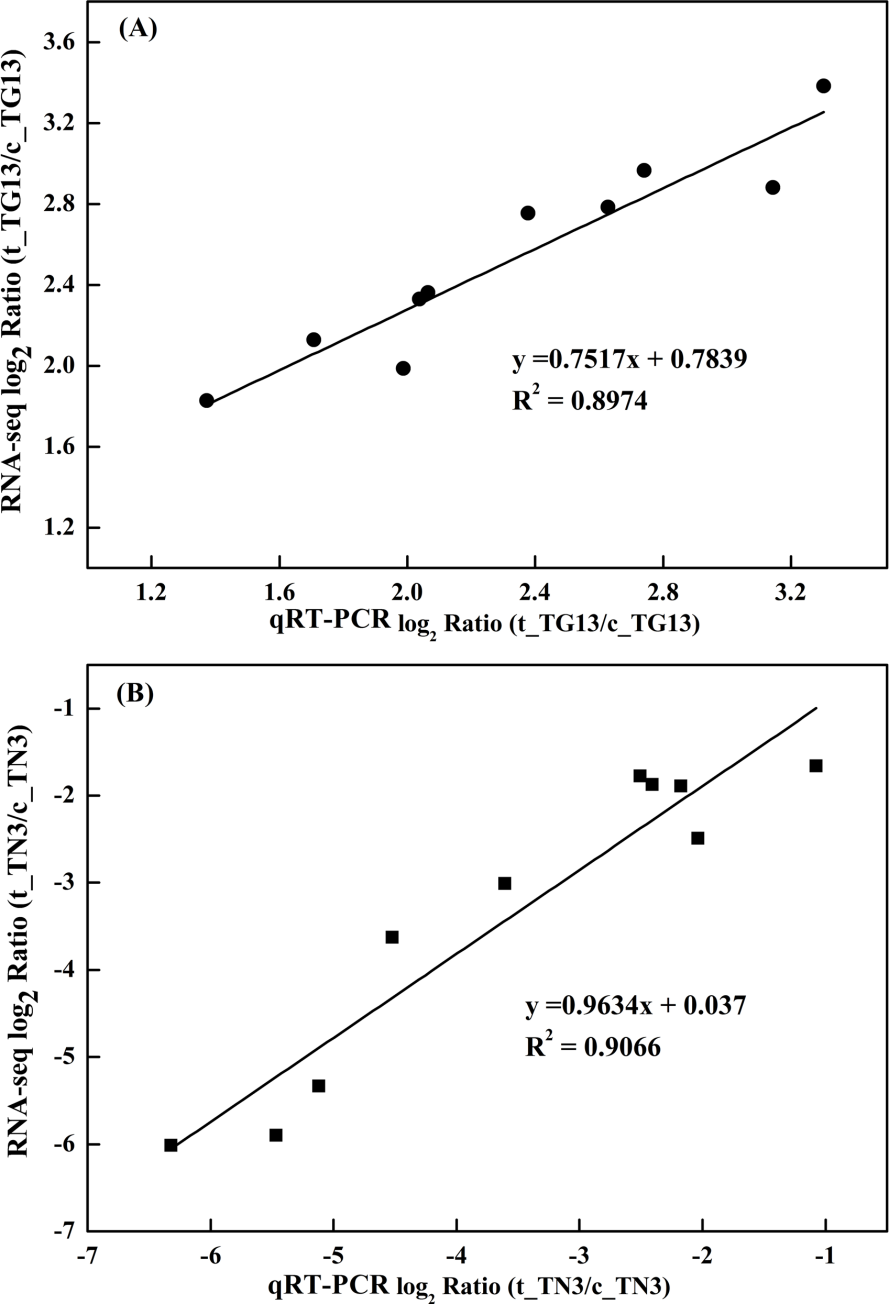
qRT-PCR validation of ten randomly selected differentially expressed genes identified by Illumina high throughput RNA-sequencing. (A): *Rhodococcus* sp. TG13; (B): *Rhodococcus* sp. TN3.

To validate the results of RNA-seq, qRT-PCRs were performed on 10 genes with different expression profiles for strains TG13 and TN3, respectively. As shown in Fig 6, the RNA-seq data agreed well with the qRT-PCR data and Pearson correlation coefficient values were 0.947 and 0.952 (P < 0.0001), respectively. Although the few differences that are often observed between qRT-PCR and Illumina transcriptome sequencing results, the trends of up- or down-regulated transcription of the 10 genes by qRT-PCR analysis with three independent biological replications were consistent with those of DEGs expression profiling analysis, which indicated that the reliability of the RNA-seq results (S9 Table).

## Discussion

In the present study, the biphenyl/PCB-degrading bacterial strain TG13 entered into the VBNC state after 91 days maintaining in AOM at 4°C, and it could be resuscitated from VBNC state by incubating in LB medium at 30°C. Notably, it is practically possible (benzylpenicillin-pre-treated VBNC cells) to confirm that the increases in culturability is due to the resuscitation of VBNC cells [17]. Indeed, the ability to be resuscitated under permitting conditions is an important characteristic of VBNC cells, and resuscitation of the VBNC state by returning to favourable conditions have been already reported [10, 17, 18]. However, strain TN3 entering the VBNC state required a much longer period of time (163 days) under the same incubation conditions. Moreover, the strain TN3 was unable to resuscitate from VBNC state by a simple reversal of unfavourable factors, and resuscitation occurred after addition of Rpf. One assumption is that VBNC cells of the strain TN3 may have entered in a resistant state for a long time before death. The number of cells which were able to resuscitate form the VBNC state decreased with time delaying in entering favourable environmental conditions [34]. Another hypothesis is that the resuscitation method described here was not the unique approach for recovering the culturability of VBNC cells of the strain TN3. Perhaps much more approaches should be taken to examine whether resuscitation of VBNC cells occurs. Indeed, some bacteria could not return to their culturable state after elimination of the stress factors, and requires a variety of environmental or chemical stimuli for recovery [35, 36]. The resuscitation process may involve “scout” cells inducing the growth of the remaining dormant cells [37]. Resuscitation requires restoration of many cellular functions, leading finally to the reacquisition of culturability [38]. Mukamolova et al. [39] suggested that several actinobacteria resuscitated by the role of a group of extracellular bacterial proteins known as Rpfs. It has been reported that Rpfs possessed muralytic activity which was probably responsible for the resuscitation function [39]. Although several hypotheses explaining Rpfs action mechanism had been published, the mechanism of Rpfs function remains to be uncovered [39, 40]. Senoh et al. [41] demonstrated that resuscitation of *V*. *cholera* could be achieved by coculture with eukaryotic cells. Obviously, the methods for induction and resuscitation of VBNC cells varied with various bacterial species.

In addition, this study was undertaken to begin elucidating the gene expression and regulation that might underlying the formation of VBNC state. Intriguingly, in some ways, the results from TG13 were in agreement with TN3, which indicated that down-regulated genes mainly encoded hypothetical protein, NADH dehydrogenase subunit, catalase, oxidoreductase (S2 and S3 Tables). The functions and biological pathways of DEGs related to the formation of VBNC state in the strains TG13 and TN3 were compared by enrichment analysis. Analysis of GO functional category enrichment demonstrated that significant enrichment terms of down-regulated genes in the strain TG13 were related to oxidoreductase activity (GO:0016491) and cofactor binding (GO:0048037). The genes which were most significantly up-regulated represented functional enrichment in protein metabolic process (GO:0019538), coenzyme binding (GO:0050662) and cell (GO:0005623) in each of the three main categories, respectively (S1 Fig). However, the function of down-regulated genes in the strain TN3 covered a comprehensive range of GO categories, “energy derivation by oxidation of organic compounds”, “RNA binding” and “intracellular” were predominant in each of the three main categories, respectively (Fig 4B). Notably, the terms of “response to oxidative stress”, “oxidoreductase activity” were significantly enriched which were consistent with the results of GO functional category enrichment in the strain TG13. Similar observation was reported by Chattopadhyay et al. [20] who suggested that oxidative stress response in bacteria is associated with cold stress. Kong et al. [18] already noted that cold-shocked cells of *Vibrio vulnificus* was peroxide sensitive due to loss of catalase activity. Meanwhile, results from the study of Smith and Oliver [42] confirmed the role of catalase in the VBNC state of *V*. *vulnificus* at low temperatures. The decreased catalase activity resulted in the cells that were unable to defend against the hydrogen peroxide present in routine media and become nonculturable. Thus, it could be inferred that cold temperature-induced loss of ability to defend oxidative stress may play a significant role in induction of the VBNC state.

Meanwhile, the results of KEGG pathway analysis indicated that “fatty acid degradation” (S2 Fig), “RNA polymerase” (S3 Fig), “citrate cycle” (S4 Fig) and “oxidative phosphorylation” (S5 Fig) were dominant pathways both in the two strains TG13 and TN3. For the pathway of fatty acid degradation, the DEGs encoding enoyl-CoA hydratase were found down-regulated in both the two strains (S2 Fig). For the pathway of RNA polymerase, the transcript of rpoA and rpoZ homologs increased in the formation of VBNC state for the strain TG13 (S3A Fig). The result also agreed with the work of Nyström [43], who found that RNA polymerase is redistributed from proliferating activities to maintenance when the environment is harsh for growth. While for the strain TN3, the transcript of rpoA decreased and no changes were found in the transcript of rpoZ (S3B Fig). Ritz et al. [44] demonstrated that *rpoA* gene encoding subunit of the transcriptional regulator sigma has constant transcript levels under various stress conditions. Meanwhile, evidence has been obtained to show that elevated expression of *rpoS* gene played a key role in enhancing stress resistance [10]. Combining the previous studies with this study, it is interesting to speculate that the expression of genes encoding RNA polymerase varied with various stresses and bacterial species.

In particular, the results of gene expression responses of the strain TG13 to VBNC state were similar to the bacterium *R*. *biphenylivorans* TG9^T^. The up-regulated genes related to protein modification, ATP accumulation and RNA polymerase were found in the VBNC cells, which render VBNC cells more tolerant to survive under inhospitable conditions. In comparison, all the DEGs related to the formation of VBNC state in the strain TN3 were found down-regulated. Perhaps the VBNC cells of the strain TN3 may have entered in a resistant state for a long time before death. Furthermore, DEGs enriched in metabolic pathway of citrate cycle in the strain TG13 were not in accord with that of in the strain TN3 (S4 Fig). For the pathway of oxidative phosphorylation, the DEGs encoding NADH dehydrogenase were found down-regulated in both the two strains (S5 Fig). It is notable that the exact role and molecular mechanism of the VBNC state are yet unknown and likely differ from bacterium to bacterium [7]. It has been demonstrated that the ATP level has been found to generally remain high in the VBNC cells [45], and even 2.5 times higher than the initial ATP level in the normal cells [46]. ATP accumulation in the VBNC cells might be significant for boosting the repair system to recover the cells from injury, and help cells survive longer after depletion of the primary energy source [46]. These reports are consistent with the results of the strain TG13. However, most of reports suggested that the energy requirements of VBNC cells were lowered due to decreased levels of respiration rates and macromolecular metabolism [7, 10, 16]. Lindbäck et al. [45] indicated that the ATP concentration is lower in VBNC cells than in growing cells. These findings are consistent with the results of the strain TN3 which demonstrated that down-regulated genes were mainly involved in “oxidative phosphorylation” and “carbon metabolism” because of minimal biochemical metabolism (bold in S7 Table). By analogy to the report of Kong et al. [18], our findings verified that a single underlying mechanism impossibly exists for the VBNC phenomenon in all bacteria, even the same genus. Undoubtedly, the inducers of the VBNC response vary from species to species. Further work is needed to reveal the molecular mechanisms underlying the VBNC state of various bacteria under varied environmental stresses.

## Conclusions

This study has clarified that *Rhodococcus* sp. strains TG13 and TN3 could enter into the VBNC state under oligotrophic and low temperature conditions, and could recover culturability under different recovery conditions. Gene expression variations in the VBNC response indicated that cold-induced loss of ability to defend oxidative stress may play an important role in induction of the VBNC state. Meanwhile, the results verified that molecular mechanism underlying the VBNC state differ from bacterium to bacterium. It remains to seen whether other pollutant-degrading bacteria could enter into the VBNC state, which would be useful for better understanding their decreased activities in the practical environmental bioremediation.

## Supporting Information

S1 Fig GO enrichment of up-regulated genes in t_TG13 and c_TG13 libraries. The y-axis denotes the number of genes in a category. The number above the bar denotes the percentage of a specific term of genes in the main category. The significant enriched GO terms were presented with Corrected P-value < 0.05.
(DOC)

S2 Fig Differentially expressed genes enriched in metabolic pathway of fatty acid metabolism in *Rhodococcus* sp. strains TG13 (A) and TN3 (B). TN3. The red and the green boxes indicate genes with up-regulated and down-regulated, respectively.
(DOC)

S3 Fig Differentially expressed genes enriched in metabolic pathway of RNA polymerase in *Rhodococcus* sp. strains TG13 (A) and TN3 (B). The red and the green boxes indicate genes with up-regulated and down-regulated, respectively.
(DOC)

S4 Fig Differentially expressed genes enriched in metabolic pathway of citrate cycle in *Rhodococcus* sp. strains TG13 (A) and TN3 (B). The red and the green boxes indicate genes with up-regulated and down-regulated, respectively.
(DOC)

S5 Fig Differentially expressed genes enriched in metabolic pathway of oxidative phosphorylation in *Rhodococcus* sp. strains TG13 (A) and TN3 (B). The red and the green boxes indicate genes with up-regulated and down-regulated, respectively.
(DOC)

S1 Table qPCR primers used in this study. (XLS)

S2 Table Gene expression value of annotated genes. (XLS)

S3 Table Differentially expressed genes. (XLS)

S4 Table Genes > 20 fold up- or down-regulated. (XLS)

S5 Table Gene Ontology function of differentially expressed genes. (XLS)

S6 Table GO enrichment analysis of up and down-regulated genes in t_TG13 versus c_TG13 cultures. (XLS)

S7 Table KEGG pathway enrichment of differentially expressed genes. (XLS)

S8 Table KEGG enrichment analysis of up and down-regulated genes in t_TG13 versus c_TG13 cultures. (XLS)

S9 Table qRT-PCR validation of transcriptomic profiles. (XLS)
