# Supplementary figures and images for: Induction of Viable but Nonculturable State in Rhodococcus and Transcriptome Analysis Using RNA-seq
Source: PLoS One. 2016 Jan 25;11(1):e0147593. doi: 10.1371/journal.pone.0147593 (PMC4725852; doi:10.1371/journal.pone.0147593)

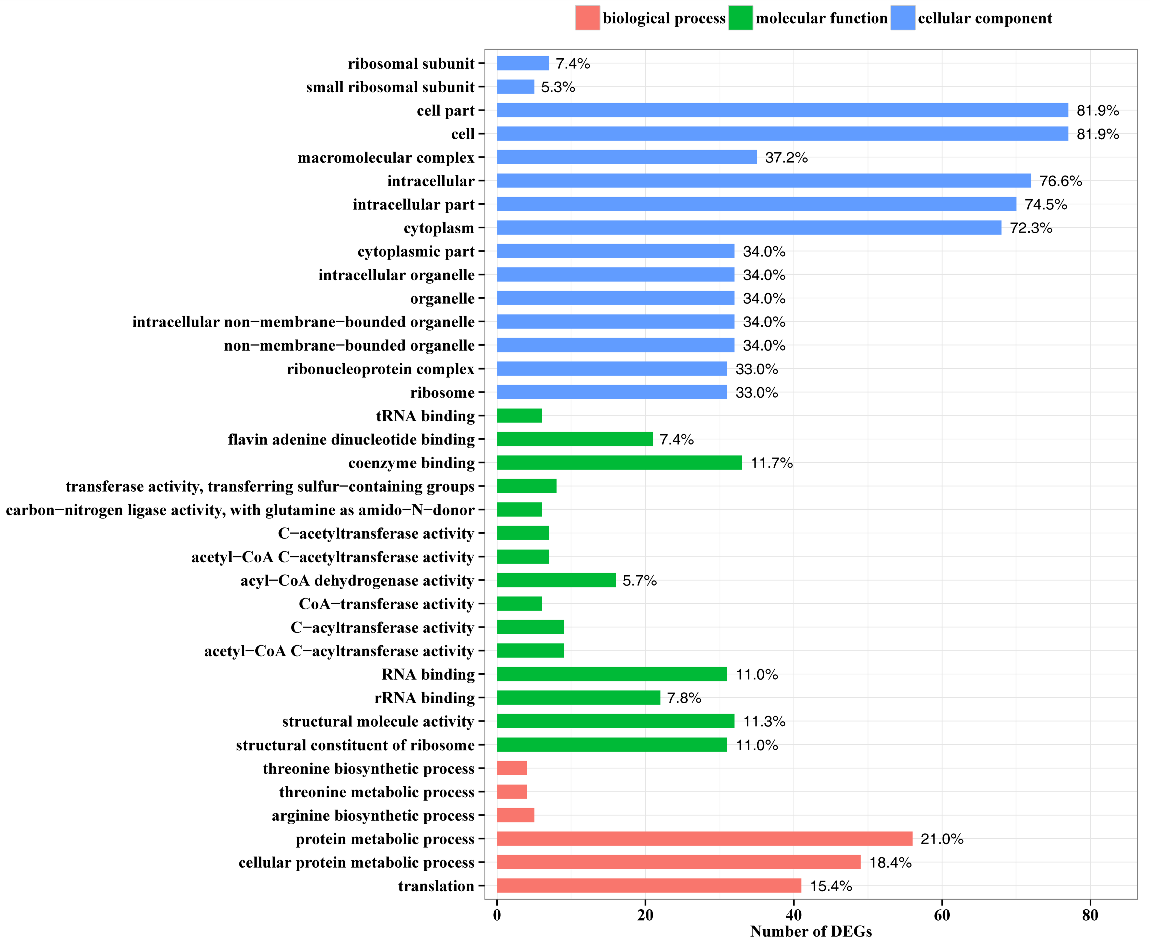


**S1 Fig**

Supplement: S1 Fig — The y-axis denotes the number of genes in a category. The number above the bar denotes the percentage of a specific term of genes in the main category. The significant enriched GO terms were presented with Corrected P-value < 0.05. (DOC) [file pone.0147593.s001.doc]

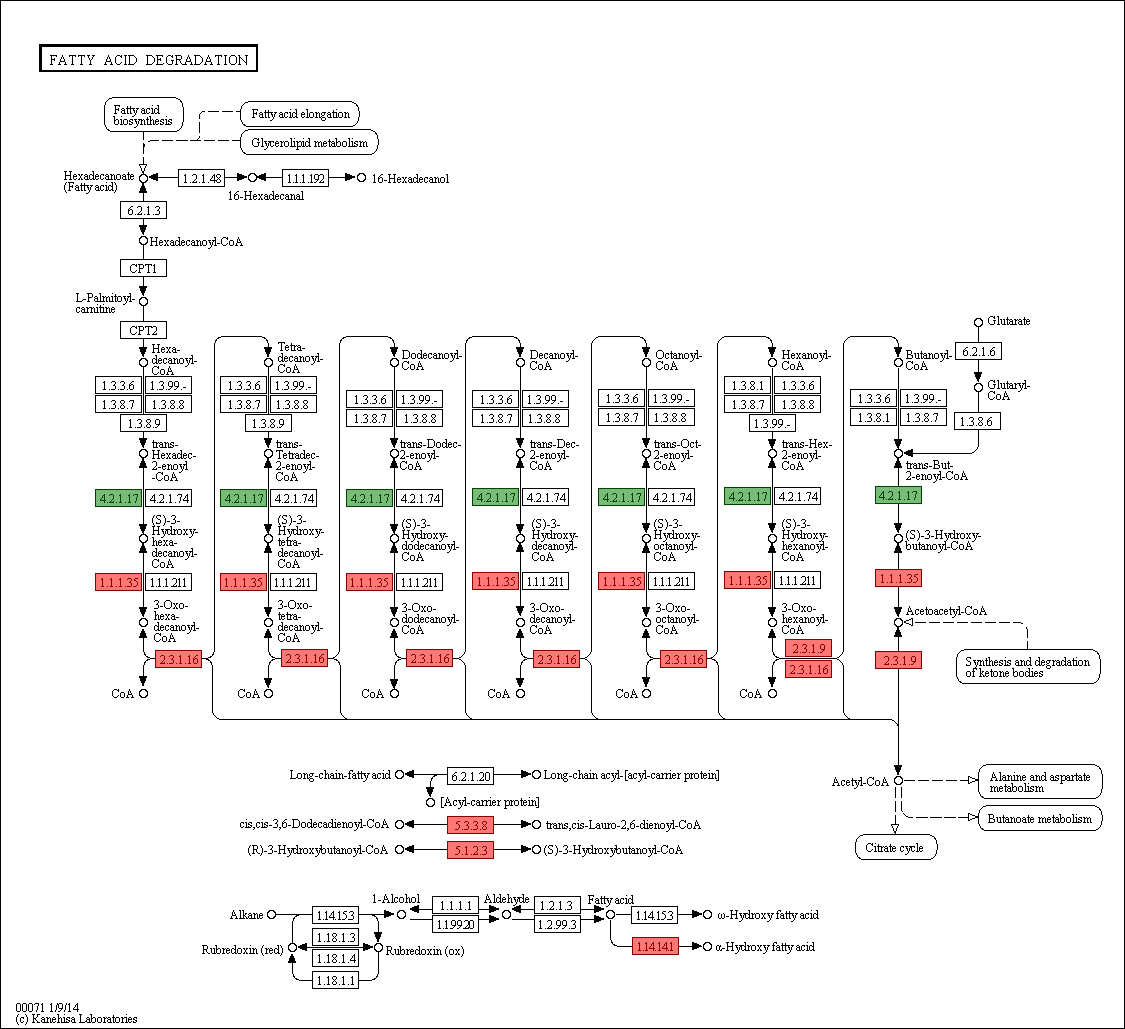


**(A)**


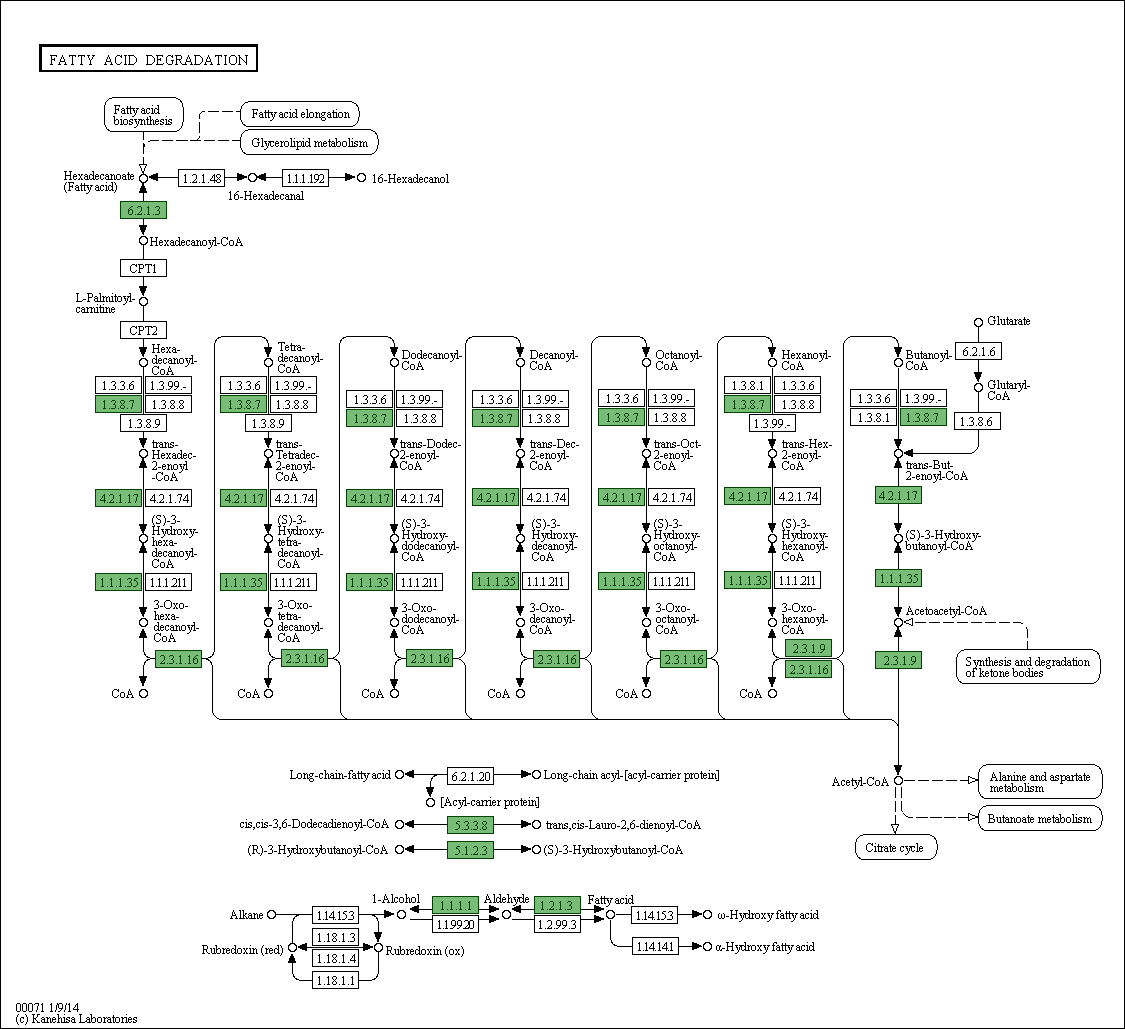


**(B)**

**S2 Fig**

Supplement: S2 Fig — TN3. The red and the green boxes indicate genes with up-regulated and down-regulated, respectively. (DOC) [file pone.0147593.s002.doc]

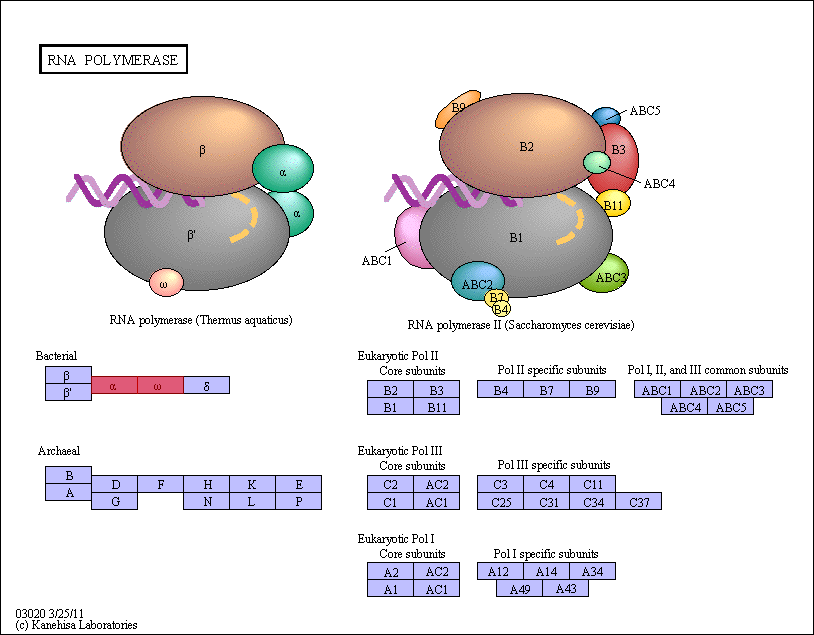


**(A)**


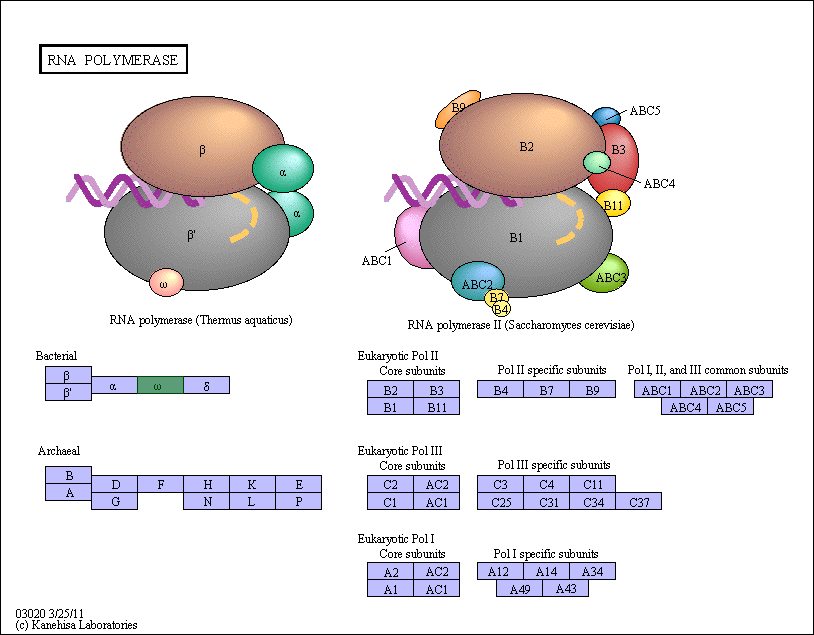


**(B)**

**S3 Fig**

Supplement: S3 Fig — The red and the green boxes indicate genes with up-regulated and down-regulated, respectively. (DOC) [file pone.0147593.s003.doc]

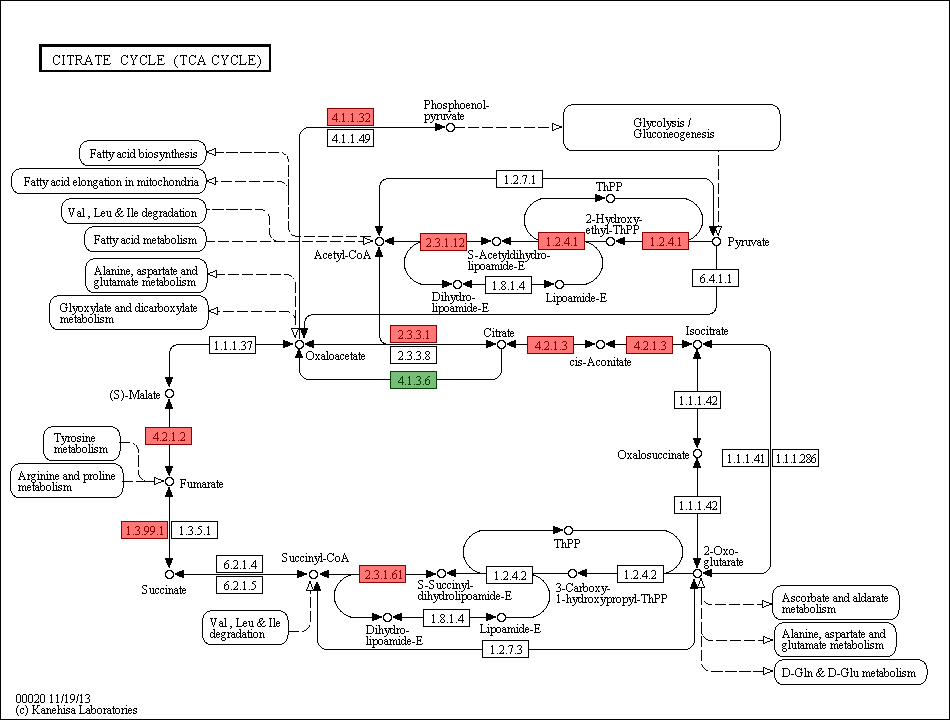


**(A)**


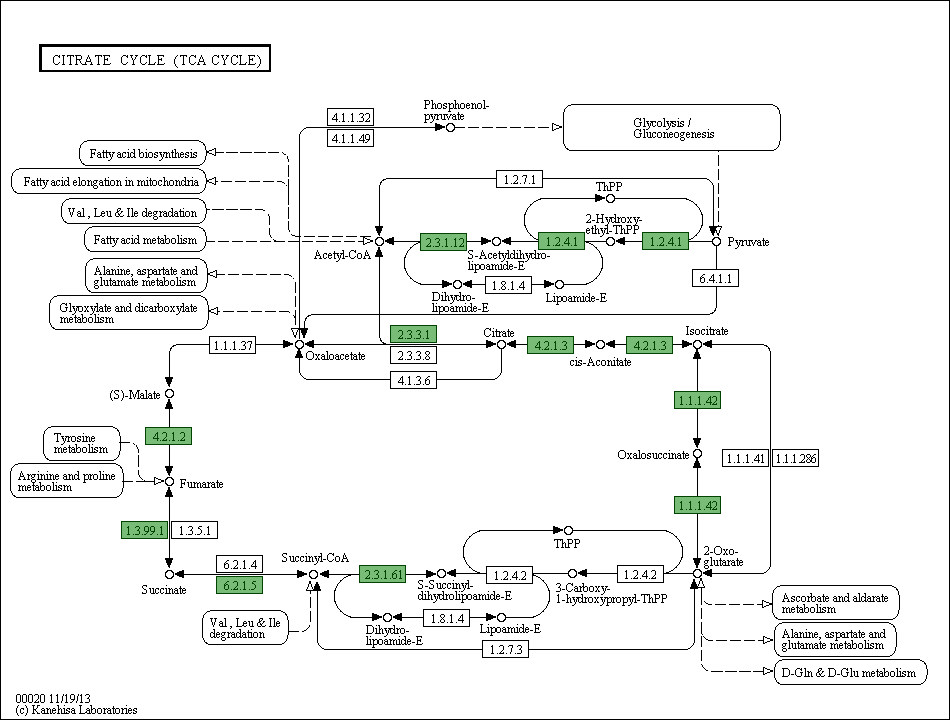


**(B)**

**S4 Fig**

Supplement: S4 Fig — The red and the green boxes indicate genes with up-regulated and down-regulated, respectively. (DOC) [file pone.0147593.s004.doc]

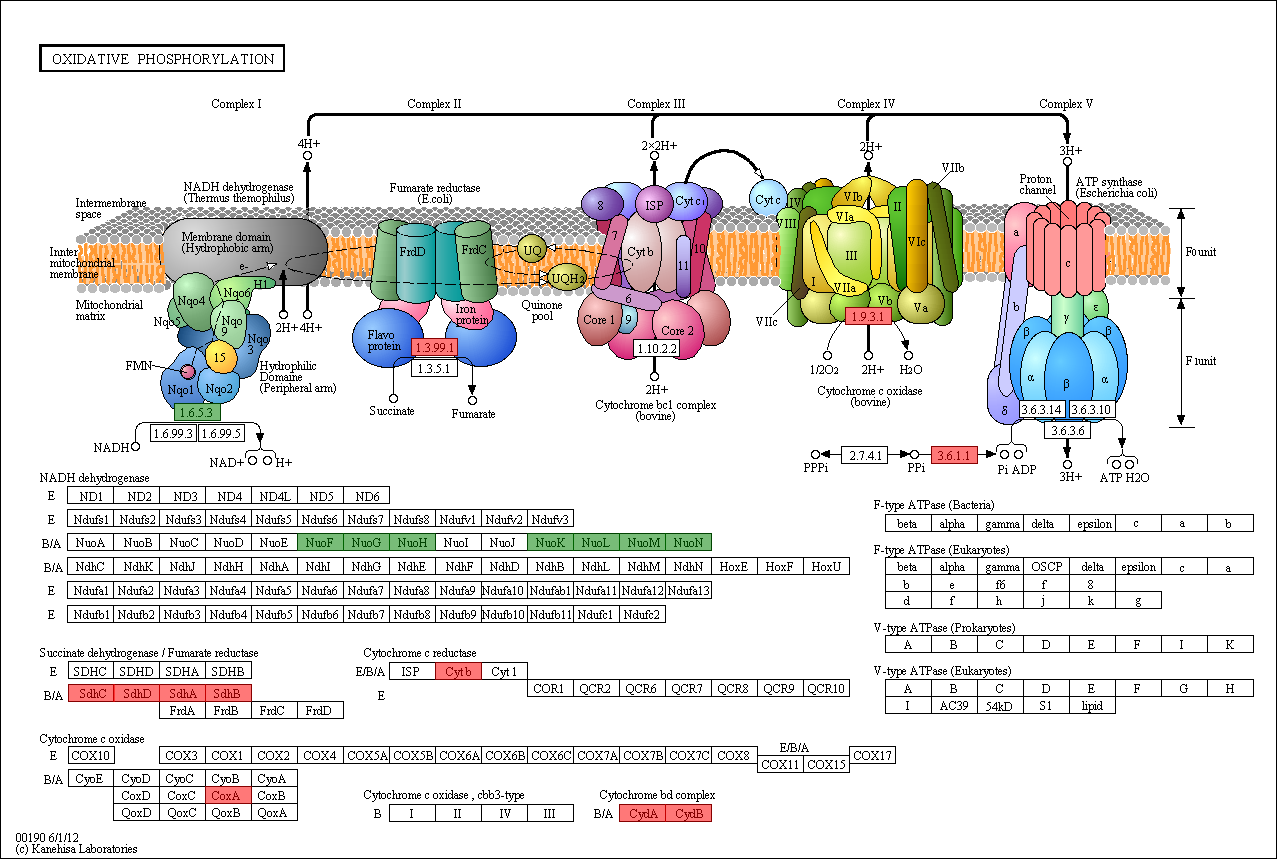


**(A)**


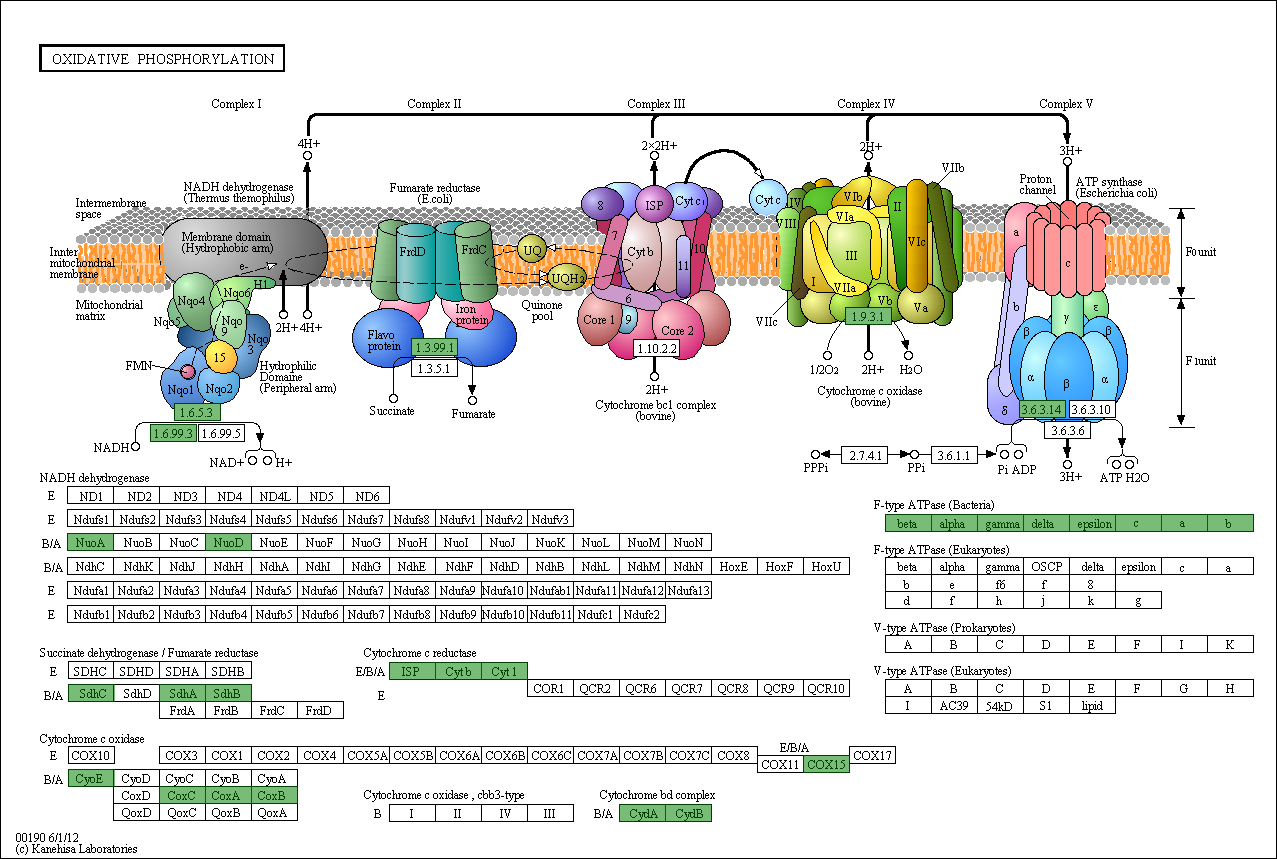


**(B)**

**S5 Fig**

Supplement: S5 Fig — The red and the green boxes indicate genes with up-regulated and down-regulated, respectively. (DOC) [file pone.0147593.s005.doc]
